# Remote Working and Work Effectiveness: A Leader Perspective

**DOI:** 10.3390/ijerph192215326

**Published:** 2022-11-20

**Authors:** Grzegorz Kowalski, Katarzyna Ślebarska

**Affiliations:** Institute of Psychology, University of Silesia in Katowice, 40-007 Katowice, Poland

**Keywords:** effective leader, leader perspective, remote working, work effectiveness, working from home

## Abstract

Currently, job duties are massively transferred from in-person to remote working. Existing knowledge on remote working is mainly based on employees’ assessment. However, the manager’s perspective is crucial in organizations that turned into remote work for the first time facing sudden circumstances, i.e., SARS-CoV-2 pandemic. The main aim of our study was to analyze remote work effectiveness perceived by managers (N = 141) referring to three crucial aspects, i.e., manager, team, and external cooperation. We assumed the perceived benefits, limitations, and online working frequency as predictors of remote work effectiveness. Further, we analyzed the possible differences in remote work perception referring to different management levels (i.e., middle-level and lower-level). Our findings revealed a significant relationship between the benefits and effectiveness of managers and external cooperation, specifically among lower-level managers. Limitations, particularly technical and communication issues, predicted team and external cooperation effectiveness. The results showed remote work assessment as being socially diverse at the management level.

## 1. Introduction

Currently, remote work has become a crucial organizational tool that enables effective performance in the increasingly competitive global market. Although working outside of the office has already been available, this form of performing job duties seems mainstream in modern organizations. Due to the SARS-CoV-2 pandemic, 14.2% of employees in Poland changed their current way of performing professional duties to a remote mode. Almost every sixth employee in the public sector and every twelfth in the private sector worked remotely [1]. 85.6% worked remotely for five days a week, and 64% were likely to perform their professional duties remotely even after returning to the work office, especially since 44% of employees declared that their efficiency at home did not decrease [2]. Half of them indicated that sufficient work outside of the office was performed mainly for two days, and every seventh employee pointed out three remote working days.

Since the COVID-19 pandemic, many studies have been conducted on various aspects of remote working from the employees’ perspectives [3,4,5,6,7]. Generally, employees find working from home productive, albeit managers are often concerned about maintaining job performance at least on the same level as office work [8,9]. Thus, it seems crucial to look at how managers at different levels of management perceive the introduction of remote working on an unprecedented scale since they are responsible for organizing and controlling the employees’ work [10]. We decided to use managerial perception as previous research has proved the usefulness of subjective performance measures and their similarity with objective internal performance [11,12,13]. This study aimed to determine how managers rated the effectiveness of their own work and how they assessed the effectiveness of their team and external collaboration while performing their job duties remotely.

### Literature Review and Hypotheses Development

Managers’ effectiveness has been defined as the impact of managers on the fluent functioning of an organization [14]. They can manage effective performance by using optimal acquisition and utilization of internal and external resources, i.e., human, financial, and instrumental resources. Since the managerial role is crucial in obtaining effective workflow and outcomes, this study was focused on managers’ perspectives.

Managers have different needs depending on their status [15]. Most often, the structure of managers in an organization consists of three levels [16,17]. The first one is top management which assumes top managers with most power, authority, and responsibility. The managers at this level define the company’s strategy, vision, and mission. They represent the company externally and visualize and define the company’s future. Top management is also responsible for dealing with the groups or individuals who may have different interests or intentions that do not have to align with the company’s interests. Their role is to unite or convince them that the interest of the organization stands above everything and is not in conflict with their actions [18]. The second level, namely middle management, is the one that sets the goals to achieve the organization’s strategy. Middle managers are tasked with communicating and implementing the plan received from top management [19]. They indicate organizational roles, and they work mainly with the low management. Thus, they rarely have contact with first-line workers. [20]. At the lowest level of the managerial hierarchy, lower-level managers usually have the most direct and frequent contact with front-line employees. As a result, low managers can significantly impact work effectiveness [21] since they operate and plan in the short term. They usually do not have the power to implement their own initiatives that can change the strategic goals [19,22]. Nevertheless, to ensure the stable functioning of the organization in unstable circumstances (e.g., at the time of the pandemic), they play a crucial role as first-line leaders. Therefore, the main objective of our study was the assessment of how managers with direct contact with subordinates (i.e., low- and middle-level managers) perceived work effectiveness.

The environment in which an organization finds itself is volatile, and managers at all levels should be open to change. Increased performance and job satisfaction from the perspective of individual employees are reported in trade journals [23] and academic sources [24]. However, the relationship between remote working and performance has not been well established from the managers’ perspective [11,12,13,25,26]. Virtual working, including working from home, comprises different benefits, e.g., saving time and other expenses, integrating the work of specialized employees, and expanding external co-operation. There is abundant research on the benefits and limitations of remote working [27]. The most common benefits include no commuting, reduced distraction, work–life balance and increased work flexibility, creativity, and motivation [28,29]. In addition, many studies have shown increased productivity [30,31]. Research indicates that proximity to co-workers often leads to wasted time and decreased productivity. The increased efficiency of employees in remote working is due to the lack of distractions present in the office [32]. On the other hand, employees indicate that the most significant disadvantage of remote work is the lack of non-work-related contacts [33], even though they can contact others via information and communication technologies (ICTs) [34]. Although Gibbs, Mengel, and Siemroth [27] emphasized that productivity depended on the worker’s characteristics, and measured employee productivity, the employees were able to maintain similar or slightly lower levels of output during work from home. Besides its positive aspects [30,35], existing research indicated a number of challenges generated by remote work, such as work–home interference, ineffective communication, procrastination, and loneliness.

As mentioned above, there are many advantages of remote forms of performing job duties, and several limitations that result in work outcomes and collaboration [31]. The responsibility of managing the remote work of employees rests with managers, particularly first-line managers and team leaders. Therefore, we assumed that the perceived effectiveness of remote work was connected with the experienced benefits and limitations (*cf.* Hypothesis 1). Moreover, different management levels, i.e., middle- and lower-level managers, might perceive remote work differently (*cf.* Hypothesis 2).

**Hypothesis 1:** 
*The perceived benefits, limitations, and frequency of remote work are related to the remote work effectiveness perceived by lower-level and middle-level managers.*

**Hypothesis 2:** 
*The perceived remote working conditions differ between lower-level and middle-level managers.*

## 2. Materials and Methods

### 2.1. Participants and Procedure

To evaluate the effectiveness of remote work, we recruited employees from one of the largest enterprises in Poland. The companies that provided data belong to one of Poland’s largest capital groups in the energy sector. The survey covered the executive staff of three companies employing 234 middle- and lower-level managers (68 women and 166 men). A total of 29% were middle-level managers. The survey mainly addressed managers who had worked remotely/hybrid since the beginning of the COVID-19 pandemic. Two of the three companies surveyed previously could use remote working, but no more than two days per month. One company did not have remote working in operation. A vast majority of the managers were college-educated employees. As a result of the COVID-19 pandemic, all companies included in the survey had started remote working with the possibility of hybrid working. In the interests of employees, it was recommended that all individuals who were able to perform their duties (i.e., had the appropriate equipment) and agreed to work remotely took advantage of this opportunity.

We focused explicitly on the management staff during recruitment, i.e., department executives. Overall, the sample comprised 141 participants, including 18.7% middle management and 81.3% lower management. A total of 71% of participants were male, which reflects a male predominance in the real structure of the labor market and the share of males in the total number of employed managers in Poland [36]. All respondents were highly skilled and educated, mainly in the engineering field.

This cross-sectional study was based on anonymized employee data selected from the organizational resources. No person-related data were collected to ensure the anonymity of the study. The respondents received a link that directed them to the survey located on the company intranet. Participation was voluntary and free of charge. The participants were informed of the voluntary nature of participation in the study and the anonymity of data collection, i.e., their data would be analyzed collectively, and no personal information would be shared. They were assured that there were no wrong answers and that all of their opinions were important. Prior to participation, the respondents provided oral consent to participate in the study and were informed about the possibility of withdrawing from the study. All employees were aged 18 or older and completed their duties remotely from home.

### 2.2. Measures

Work effectiveness was assessed with three items related to different remote work effectiveness dimensions, i.e., the respondents were asked to assess the effectiveness of their own work, of the team, and of the co-operation with other business areas. All items required the participants to rate the extent to which they perceived work effectiveness (sample question: “Taking everything into consideration, how do you rate your work effectiveness as a whole?”) in all dimensions using a 5-point scale from 1 (ineffective) to 5 (very effective). Each dimension contained one-item measures. Using single-item measures is effective and more favorable in some respects than using multiple-item measures [37]; e.g., single-item measures are easier to understand by management, are completed more quickly, and require less effort. Higher scores indicated a higher level of perceived effectiveness in each dimension. The reliability of the scale comprising all three items in the current study was considered good, with Cronbach’s α = 0.8.

Benefits were measured using the one-item scale to assess perceived advantages of remote work with multiple-choice answers (sample categories: possibility to gain technical skills, on-task concentration, organized home life, and work economy). The list of chosen benefits was evaluated in terms of subjective fulfillment of criteria for remote working benefits by using competent judges. Benefits were defined as positive aspects, advantages, or profits gained from remote work. We asked five professionals, who were psychologists and managers, to evaluate the set of benefits on a 5-point scale (1 = does not refer to the dimension; 5 = fully refers to the dimension) and inspected the judges’ congruency concerning individual ratings (congruency index = 0.95). The ten benefits of remote work were positively verified by all five judges and were included in the study. The respondents reported the perceived benefits by checking them on a prepared list. The sum of selected benefits indicated the level of perceived benefits gained from remote work. In other words, a higher score indicated a larger number of benefits of remote work.

Limitations were measured with multiple-choice answers using a three-item scale assessing three dimensions of perceived disadvantages of remote work (i.e., organizational, technical, and social limitations). Limitations were defined as work aspects that limit the quality or achievement during remote work. The given limitations were verified by competent judges (congruency index = 0.93) and were introduced to the study. The overall-limitations measure was obtained by summing reported limitations from the possible ten statements which tap the various remote job facet (e.g., organizational, technical, and social issues). Higher scores indicated a higher level of limitations of remote work. The reliability of the scale comprising all three items in the current study was satisfying, Cronbach’s α = 0.7.

The respondents indicated the number of days of remote work per week to gain satisfactory team effectiveness, and the number of days of remote work per week to gain satisfactory management effectiveness. They rated on a scale between one to five working days.

## 3. Results

Table 1 displays means, standard deviations, and correlations for the study variables.

The management position (i.e., lower-level and middle-level management) was negatively related to the perceived benefits (*p* ≤ 0.05) and work effectiveness (*p* ≤ 0.05), and positively associated with social limitations (*p* ≤ 0.05).

In the first step, a regression analytical procedure was conducted to test the interaction between remote work conditions, i.e., benefits, limitations, online working frequency, and remote work effectiveness (*cf.*, hypothesis 1). The regression model explained 37% of the variance in managers’ effectiveness (F(2, 134) = 17.94, *p* < 0.001), 31% of the variance in team effectiveness (F(2, 134) = 15.89, *p* < 0.001), and 37% of the variance in external co-operation efficacy (F(2, 134) = 13.45, *p* < 0.001). The managers’ position was dummy-coded and contrasted with “lower-level managers” and “middle-level managers”. The results are given in Table 2.

Table 2 shows the regression analysis of the relationship between dependent variables, i.e., manager effectiveness, team effectiveness, co-operation effectiveness, and predictors. Leader effectiveness was negatively related to a managerial position. The managers’ position was dummy-coded (0 = lower-level management; 1 = middle-level management). As shown in Table 2, middle-level managers perceived the effectiveness of their work as lower (*β* = −0.15, *p* < 0.05). Positive relationships were observed between the perceived benefits of remote work (*β =* 0.14; *p* < 0.05), online working days (*β =* 0.34; *p* < 0.01), and managers’ effectiveness. The same regression analyses were conducted for team effectiveness and relations with the external environment. Team effectiveness perceived by managers was negatively related to the experienced technological limits during remote working (*β =* −0.20; *p* < 0.05) and positively related to the number of online working days (*β* = 0.33; *p* < 0.05). The results showed that co-operation effectiveness was negatively related to the perceived technological limitations (*β* = −0.18, *p* < 0.01), positively associated with the perceived benefits (*β* = 0.22, *p* < 0.01), and positively associated with the frequency of remote work of managers (*β* = 0.09, *p* < 0.05) and the team (*β* = 0.32, *p* < 0.05).

Secondly, we assessed the significance of mean differences in remote work conditions perceived by lower-level and middle-level managers (*cf.* hypothesis 2). The scores were normalized to a 0 to 1 range. We applied a Mann-Whitney U test that showed significant differences in the level of the perceived benefits of remote work between these groups (U = 642.50, *p* = 0.04). Middle-level managers perceived lower benefits (*M* = 0.29) compared to lower-level managers (*M* = 0.38). Analyzing the online work limitations, we found significant differences in the level of social limits (U = 1138, *p* = 0.02) and work effectiveness, (U = 519, *p* = 0.02) between the groups. Middle-level managers reported a higher level of social limits (*M* = 0.30) compared to the lower-level managers (*M* = 0.22). However, lower-level managers assumed themselves as more effective (*M* = 4.37) compared to middle-level managers (*M* = 3.95).

**Figure 1:**
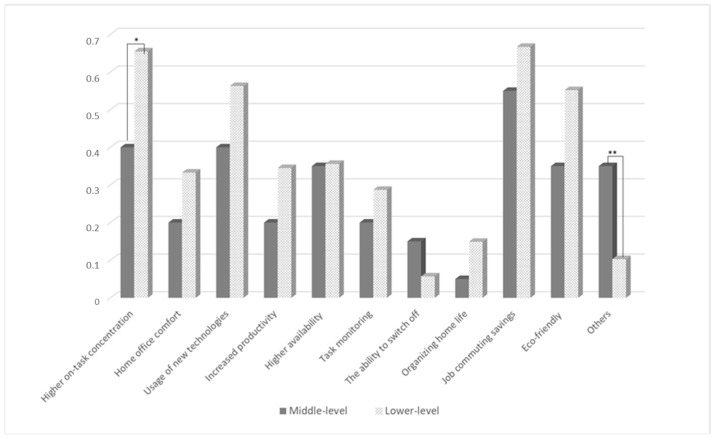
Remote work benefits perceived by lower- and middle-level managers. Notes. * *p* < 0.05; ** *p* < 0.01.

**Figure 2:**
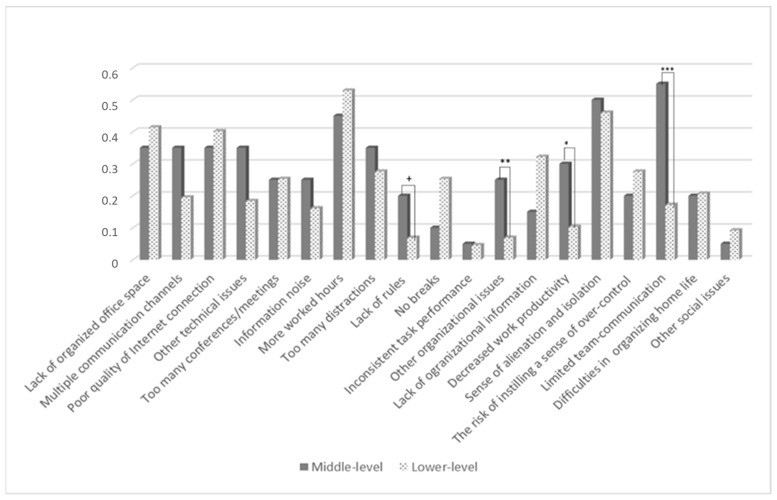
Remote work limitations, perceived by lower- and middle-level managers. Notes. * *p* < 0.05; ** *p* < 0.01; *** *p* < 0.001; + *p* < 0.10.

Based on the Mann-Whitney U test results, Figure 1 and Figure 2 present the benefits and limitations perceived by the analyzed groups in more detail. The *p*-value demonstrates significant means differences between the low- and middle-level management. 

We further tested the relation between the specified benefits (i.e., on-task concentration), limitations (i.e., lack of rules, decreased work productivity, poor communication), and perceived work effectiveness that significantly differentiated managers on different management levels. A Mann-Whitney U test showed that the communication issue and perceived own work effectiveness revealed a differential pattern (U = 1993.50, *p* = 0.02). In other words, managers who reported poorer communication as a limitation of remote working had a lower level of the perceived own work effectiveness than those who indicated no communication issues. A significant difference was observed in work effectiveness referring to perceived productivity (U = 1882.50, *p* = 0.001). A lower level of managers’ effectiveness was shown in managers who experienced lower productivity.

Although the lack of rules did not significantly differentiate own work effectiveness, the perceived effectiveness of co-operation with the environment was significantly different for managers who “suffered” more from a lack of rules than those who did not complain (U = 1099, *p* = 0.03).

On-task concentration reported by managers was significant in differentiating their work effectiveness (U = 1475, *p* = 0.001) indicating that managers who reported on-task concentration as a remote work benefit perceived better work effectiveness.

## 4. Discussion

The COVID-19 virus outbreak has made many people work from home on an unprecedented scale, especially in business sectors where employees had not had an opportunity to work remotely before. Consequently, we argued the necessity of conducting research to confirm the effectiveness of remote work in this unique context, particularly from the managers’ perspective.

First, we examined the role of the perceived benefits, limitations, and online working frequency in maintaining high work effectiveness in three dimensions (i.e., manager, team, and external collaboration levels). Our findings showed benefits as significant predictors of perceived manager and co-operation effectiveness. The more benefits managers reported, the more effective they felt at work. Therefore, activating the available strengths of remote work empowers organizational resources and work effectiveness. Available communication devices allow quicker performance of the tasks e.g., organizing and attending work meetings online is faster and easier compared to organizing face-to-face contacts [38]. This relationship mainly concerns lower-level managers. From the managers’ perspective, the benefits were not as important in predicting the team’s effectiveness. The results indicated significant relationships between technical limitations and effective remote work in team and external collaboration. Technical issues were perceived as lowering work effectiveness, independently of the manager’s management level (i.e., middle-level and lower-level).

Further analysis demonstrated the differences in the perception of work effectiveness among managers at different levels of management (i.e., lower-level and middle-level management). In the context of remote working introduced on such a large scale during the COVID-19 pandemic, our findings highlight that, on the one hand, increased effectiveness and perceived benefits can be observed. On the other hand, they are not at the same level depending on the management role connected with social interactions.

Our findings offer managers a new lens to view the advantages/disadvantages of working from home. Generally, employees’ lack of social interactions is perceived as a disadvantage [34]. Nevertheless, this study proposes an alternative view of telecommuting that can boost performance as a result of improving technical support and minimalizing unnecessary distractions. Although, Allen, Golden, and Shockley [9] emphasized that social relationships at work can suffer as a result of excessive remote work, and care should be taken to properly manage the negative effects of weakened relationships between employees. We cannot lead to workplace loneliness which can result in lower job performance [39] as a result of informal interactions and a team cohesion decrease [7]. The results showed that the possibility of concentration on the task was evaluated higher by lower-level managers. Work that requires more on-task concentration and problem-solving is done more preferably at home, with significantly fewer distractions [29,40]. As mentioned before, lower-level managers have more frequent contact with employees than higher-level managers, and recent research suggests that calls between remote workers are more task-focused and less distracted [32,34]. Consequently, referring to perceived remote work limitations, organizational issues (e.g., lack of rules), and social issues (i.e., lower productivity and ineffective communication with employees) significantly differentiated the managers at different managerial levels. The middle-level managers suffered more from the specific remote work limitations.

By identifying differences in the managerial levels in the perceived benefits and limitations, our findings shed light on a specific explanation as to why remote working is perceived more favorably by lower-level managers. Therefore, our empirical studies on how social implications of remote working can affect work effectiveness [32] indicated that a lack of distractions can increase workers’ effectiveness while working from home. We do not argue that the effectiveness of the remote mode is only due to employees’ lack of distraction in the home office. The perceived benefits and technological issues are also related to work effectiveness. An understanding of how managers perceive remote work and its effectiveness at different managerial levels and the discrepancy in the perception of benefits and limitations is crucial for understanding remote work effectiveness, especially since remote working offers indisputable convenience, which will contribute to its expansiveness in the organizational setting compared to the pre-COVID-19 level.

### 4.1. Limitations and Direction for Further Research

Despite the contributions we make, this study is not without limitations. First, our research did not explore the employees’ perspective or objective internal performance or work characteristics. Nonetheless, the managerial perspective is relatively rarely analyzed. Future research could explore how employee attributes and other factors such as personality or stress may shape the effectiveness of working online. Second, the sample size was comparatively small, with a male predominance, which limits the generalizability of the findings and the opportunity to explore other moderating mechanisms. Nevertheless, the sample provided sufficient statistical power to test the hypothesized relations. Next, our study was designed as cross-sectional. Considering the specificity of the sample and contextual conditions (i.e., pandemic), the cross-sectional design seemed reasonable and indicated the most significant relations. Finally, we used self-reported measures that are often the only possible way to examine one’s own perspective, such as self-perceived effectiveness in a specific context [34]. Nonetheless, there is still the need to use objective methods and include the employees’ perspective in the study. Using objective information (e.g., Key Performance Indicators or Return on Investment) could help solve this potential bias in the data in a future study.

Remote working in Poland is relatively new and introducing it on a such significant scale might provide unique experiences. Little is known about both direct and ripple effects that can bring us a widespread shift to remote work. Additionally, it would be useful to analyze the further relationship between social interactions and effectiveness by using objective measures. Further research requires more information concerning working online from a leader’s perspective. Longitudinal research would be necessary to demonstrate the development and changes of home office effects. Although the consideration of a leader’s perspective has given us new insights, avoiding a biased managerial perception of remote working as less effective is helpful. A more specific analysis of job characteristics and effectiveness can reveal conditions that are advantageous for employers and employees. Further interaction effects between remote work and HRM policies, as well as between social interactions, should be studied.

This study was conducted during the COVID-19 pandemic for the first time. In order to rule out the impact of pandemic stress and its effect on effectiveness, it is necessary to repeat the study after the epidemiological threat has ceased. If home-office information on a management level is available, and if a comparison during and after the coronavirus crisis is possible, we can learn whether COVID-19 has contributed to a substantial structural change.

Other constraints that can affect leaders and managers are those that also can be connected with the issues that are familiar from the perspective of employees. One such constraint, for instance, might be the low turnover and the intensity of hiring, which was limited. In the case of employees, a decline in efficiency can be observed, which could be partly traced to having less experience, lower tenure, or being in the process of onboarding [27].

### 4.2. Practical Implications

This study provides meaningful implications for practitioners. First, our research suggests that effectiveness can be increased by managing remote work effectively and implementing HR policies to strengthen the benefits of remote work and minimalize shortcomings, mainly in technical dimensions (e.g., poor quality of internet connections, multiple communication channels), while organizations can set hybrid working from home and observe changes in the managerial perception. However, organizations may influence the supportive practices that come to managers of all levels. Employers can offer training on improving their managing skills in remote environments. Some researchers suggest that consideration should be given to the individual adjustment of work conditions (e.g., less disciplined employees might experience more challenges during remote working). Therefore, offering them online work would be unsuccessful [34].

Researchers emphasize the great role of managers and leaders in practicing working from home. They are ought to provide adequate support in response to the needs of employees with different challenges [7,34]. Otherwise, remote working might turn out to be ineffective causing problems such as a longer time spent on projects, difficulties with training, onboarding issues, etc. We can observe that, from a management point of view, working from home reached the highest level of productivity in COVID-19 and stabilized, but this situation might not be sustainable [40].

The main concern, from a managerial perspective, often suggested about working from home is a decrease in effectiveness [8]. Thus, it can have a negative effect on how they operate at different levels of management. This study contributes to clarifying this issue and gaining a better understanding of the sources of perceived effectiveness from the perspective of managers and leaders. It can have a positive impact on the level of employees’ commitment and dedication to their companies, resulting in higher effectiveness [8].

Without a doubt, remote work has become an inherent work system, and the challenge today is to maintain or indicate maximum efficiency. Undoubtedly, the best solution is to introduce hybrid work and combine remote work with office work [23]. It is necessary to take a closer look at the characteristics of the job in question and put in place solutions to perform tasks at their best, depending on whether it is more efficient to do them at home or in the office. So far, we know that some work is done effectively at home, while other work is better done at the office.

## 5. Conclusions

This study contributes to understanding how remote working influences effectiveness from the managers’ perspective. While previous research has recognized that working online may be more effective, the role of managers has received less attention, both theoretically and empirically. Generally, managers view remote working as resulting in decreased performance and lower managerial control [8]. Our study suggests that the more benefits managers perceive, the more effective their work is assessed in different dimensions (i.e., manager, team, external co-operation). Moreover, the results indicated the difference in remote work perception depending on the management level (i.e., lower-level and middle-level management). Managers who have more contact with employees are more aware of the benefits of working remotely. Accordingly, the perceived benefits are related to a higher level of reported work effectiveness.

## Figures and Tables

**Table 1:** Means (*M)*, standard deviations (*SD*), and correlations between study variables.

Variable	*M*	*SD*	1	2	3	4	5	6	7	8	9	10
1. Position ^a^	—	—	—									
2. Online_leader	3.31	1.24	−0.12	—								
3. Online_team	3.31	1.22	−0.11	0.87 ***	—							
4. Benefits	0.35	0.13	−0.23 *	0.22 *	0.20 *	—						
5. Limitations	0.26	0.13	0.12	−0.30 ***	−0.43 ***	−0.07	—					
6. Limit_org	0.23	0.16	0.01	−0.22 **	−0.33 ***	−0.04	0.76 ***	—				
7. Limit_tech	0.33	0.19	0.08	−0.26 **	−0.34 ***	−0.05	0.82 ***	0.48 ***	—			
8. Limit_soc	0.23	0.17	0.20 *	−0.21 *	−0.32 ***	−0.09	0.74 ***	0.34 ***	0.39 ***	—		
9. Effect_leader	4.26	0.75	−0.28*	0.54 ***	0.51 ***	0.29 ***	−0.32 ***	−0.21 *	−0.25 **	−0.28 ***	—	
10. Effect_team	4.16	0.76	−0.13	0.50 ***	0.55 ***	0.10	−0.36 ***	−0.25 **	−0.35 ***	−0.22 **	0.70	—
11. Effect_co	3.96	0.81	−0.08	0.49 ***	0.54 ***	0.31 ***	−0.39 ***	−0.31 **	−0.36 ***	−0.24 ***	0.53	0.17 ***

Notes. Limit_org—limitations in the organizational dimension; Limit_tech—limitations in the technical dimension; Limit_soc—limitations in the social dimension; Online_leader—number of days of remote work to maintain high management effectiveness (per week); Online_team—number of days of remote work to maintain high team effectiveness (per week); Effect_leader—leader effectiveness; Effect_team—team effectiveness; Effect_co—external co-operation effectiveness; ^a^ Position is dummy-coded (1 = middle-level manager, 0 = lower-level manager); * *p* < 0.05; ** *p* < 0.01; *** *p* < 0.001.

**Table 2:** Hierarchical linear regression of three aspects of remote work effectiveness.

Predictor	Leader Effectiveness		Team Effectiveness		Co-Operation Effectiveness	
	*Β*	*t*	*Β*	*t*	*Β*	*t*
Position ^a^	−0.15	−2.10 *	−0.05	−0.73	0.03	0.47
Benefits	0.14	1.99 *	−0.01	−0.19	0.22	3.11 **
Limits_org	−0.03	−0.37	−0.01	−0.17	−0.08	−0.99
Limits_tech	−0.05	−0.60	−0.20	−2.29 *	−0.18	−2.21 **
Limits_soc	−0.11	−1.39	−0.01	−0.01	−0.01	−0.09
Online_leader	0.34	2.90 **	−0.14	1.02	0.09	0.70 *
Online_team	0.08	0.62	0.33	2.33 *	0.32	2.36 *
F	17.94 ***		15.89 ***		13.45 ***	
R^2^	0.37		0.31		0.37	
Adj. R^2^	0.33		0.28		0.33	

Notes. Limit_org—limitations in the organizational dimension; Limit_tech—limitations in the technical dimension; Limit_soc—limitations in the social dimension; Online_leader—number of days of remote work to maintain high management effectiveness (per week); Online_team—number of days of remote work to maintain high team effectiveness (per week); ^a^ Position is dummy-coded (1 = middle-level manager, 0 = middle-level manager); * *p* < 0.05; ** *p* < 0.01; *** *p* < 0.001.

## Data Availability

All necessary data samples are provided in the paper.
